# Supplementary material for: The Impact of IL1B rs1143634 and DEFB1 rs11362 Variants on Periodontitis Risk in Phenylketonuria and Type 1 Diabetes Mellitus Patients in a Latvian Population
Source: Diagnostics (Basel). 2024 Jan 16;14(2):192. doi: 10.3390/diagnostics14020192 (PMC10813900; doi:10.3390/diagnostics14020192)
Supplement: Supplementary file 1 [file diagnostics-14-00192-s001.zip › diagnostics-2788499-supplementary.pdf]

Supplementary Materials

Supplementary Material S1. *Silness-Löe index assessment.*

Selection of teeth for assessment	Both soft debris and mineralized deposits were evaluated on the following teeth: dd 16, 12, 24, 36, 32, 44.<br>If any of the teeth were missing, they were not substituted.
Examination and scoring	A numerical score ranging from 0 to 3 was assigned to each of the four surfaces of the teeth (buccal, lingual, mesial, and distal) based on the following scores and criteria:<br>0 Absence of plaque – the tooth surface is assessed by running a probe across a dried tooth surface adjacent to the gingiva. If the probe does not accumulate any soft debris, the region is deemed to be free of plaque.<br>1 Plaque accumulation present on the free gingival margin and adjoining tooth area – plaque can be visualized only after the application of a disclosing solution or by running a dental probe along the surface of the tooth.<br>2 There is a moderate quantity of soft debris within the gingival pocket or along the gingival margin, which is visible to the naked eye without additional tests.<br>3 There is an extensive amount of soft debris within the gingival sulcus and/or on the tooth and gingival margin.
Calculating the index	The index was initially determined for each examined tooth by summing the scores assigned to each surface and subsequently dividing the total by four. The patient's index was derived by adding up the indices for each of the six teeth and subsequently dividing the sum by six (the number of examined teeth). In the present study, decimal scores equal to or greater than 5 were rounded up, while scores less than 4 were rounded down to whole numbers.

Supplementary Material S2. *Greene-Vermillion index assessment.*

Selection of teeth for assessment	All four first molars were examined (the buccal surfaces of upper molars and lingual surfaces of lower molars).<br>In the anterior region, the labial surfaces of upper right and lower left central incisors were inspected.
Examination and scoring	The first step was to inspect the six tooth surfaces for debris (soft matter loosely attached to teeth) by gently running a probe along the tooth surface. The presence of calculus (a hard deposit found occlusal or apical to the gingival margin) was then assessed by probing lightly along the tooth crown and root surfaces.<br>The scoring system employed for the evaluation of soft debris was as follows:<br>0 Absence of debris.<br>1 Up to one third of tooth crown covered by soft debris.<br>2 Soft debris covers more than one third but less than two thirds of the tooth crown.<br>3 Soft debris covers over two thirds of the tooth crown.<br>The scoring system employed for the evaluation of calculus was as follows:<br>0 Absence of calculus.<br>1 Up to one third of tooth crown covered by supragingival calculus.<br>2 Supragingival calculus extending over more than one third but less than two thirds of the tooth crown, or the existence of isolated specks of subgingival calculus near the cervical region of the tooth.<br>3 Supragingival calculus encompassing over two-thirds of the tooth crown, or an extensive band of subgingival calculus encircling the cervical region of the tooth.
Calculating the index	The debris scores for each tooth were summed and divided by the total number of surfaces examined. The same approach was applied for obtaining the calculus score. To calculate the Greene-Vermillion Index, the mean scores of debris and calculus measurements were added up. The debris and calculus values can be 0–3, while the index values can be 0–6. In the present study, decimal scores equal to or greater than 5 were rounded up, while scores less than 4 were rounded down to whole numbers.

Supplementary Results

Supplementary Result S1. *Genotype frequencies in tested groups.*

		Minor allele homozygotes	Heterozygotes	Major allele homozygotes	Minor allele frequency
IL1B rs1143634	PKU	5	10	26	0.243902
	%	12.20%	24.39%	63.41%	
	DM	6	11	10	0.425926
	%	22.22%	40.74%	37.04%	
	Control group	7	18	35	0.266667
	%	11.67%	30.00%	58.33%	
DEFB1 rs11362	PKU	8	26	9	0.488372
	%	18.60%	60.47%	20.93%	
	DM	9	9	10	0.482143
	%	32.14%	32.14%	35.71%	
	Control group	11	30	21	0.419355
	%	17.74%	48.39%	33.87%	
